# Preoperative Ultrasonographic Assessment of the Gastric Antrum in Diabetic Patients Before Elective Surgery: A Prospective Observational Comparative Cohort Study

**DOI:** 10.7759/cureus.76014

**Published:** 2024-12-19

**Authors:** Savitri Velayudhan, Joseph Rajesh, Sivaramakrishnan Dhamotharan, Pratheeba Natarajan, Ravindra Bhat

**Affiliations:** 1 Anaesthesiology, Indira Gandhi Medical College and Research Institute, Puducherry, IND

**Keywords:** anesthesia complications, diabetes mellitus, gastroparesis, pulmonary aspiration, ultrasonography

## Abstract

Background: Diabetic patients may have neuropathy-induced gastroparesis predisposing them to aspiration. Point-of-care gastric ultrasonography (GUSG) is useful in the qualitative assessment of the antrum and quantitative prediction of gastric volume (GV) in diabetic patients. In this study, we compared the GUSG findings between fasting diabetic and non-diabetic patients in the elective preoperative setting.

Methods: A total of 220 patients were included in the study with 110 diabetic patients in the diabetes mellitus (DM) group and 110 non-diabetic patients in the non-diabetes mellitus (NDM) group. GUSG was performed in supine and right lateral decubitus (RLD) positions and qualitative grading was done. An empty antrum in both supine and RLD positions was graded 0. Fluid present in the RLD position but empty in the supine position was graded 1. The presence of solids or fluid in both supine and RLD positions was graded 2. Quantitative assessment was done by calculating the estimated GV using the measured cross-sectional area (CSA). The presence of grade 2 antrum or solids or GV >0.8 mL/kg was considered as criteria for a ‘high-risk’ antrum.

Results: Grade 2 antrum was found in 18% of patients in the diabetic group compared to 3% in the non-diabetic group. Mean CSA (5.65 cm^2^) and mean GV (34.52 mL) were significantly higher in diabetic patients. Forty-one (37.2%) patients among the diabetic patients had a high-risk antrum and a potentially higher risk of aspiration when compared to non-diabetic patients. Higher age and female gender were found to be associated with the incidence of ‘high-risk’ antrum in the univariate logistic regression model.

Conclusion: Diabetic patients have a higher incidence of grade 2 antrum, and higher CSA and GV, when compared to non-diabetic patients. Risk factors such as female gender and increasing age are associated with the high-risk antrum incidence. Further studies where objective tests are done to identify the presence of diabetic autonomic neuropathy might help determine the relationship between GUSG and aspiration risk.

## Introduction

Aspiration pneumonitis is a rare but dangerous complication associated with general anesthesia in surgical patients [1]. Patients are advised preoperative fasting to reduce the risk of this complication. Fasting guidelines are standard for all surgical populations [2]. However, there are groups of patients who are at risk of aspiration more than others. Diabetic patients may have gastroparesis, which increases the risk of aspiration during general anesthesia. The standard fasting guidelines may not be enough to ensure safe gastric residual volume (GRV). With point-of-care ultrasonography (POCUS), it is possible to image the gastric antrum and calculate the gastric volume (GV) objectively, thereby enabling us to predict the aspiration risk with better precision [3]. Based on the ultrasonographic appearance of the antrum, it can be classified into three grades, namely, 0, 1 and 2 [4]. The primary objective of this study was to compare the incidence of grade 2 antrum in diabetic and non-diabetic patients. The secondary objective was to compare the incidence of grade 0 and grade 1 antrum, cross-sectional area (CSA), and GV in both groups and predict the risk factors for the presence of a ‘high-risk’ antrum.

## Materials and methods

The study was conducted at a tertiary care institute, Indira Gandhi Medical College and Research Institute (IGMCRI), Puducherry, India, and included patients undergoing elective surgery between July 12, 2019, and July 17, 2023. Informed consent was obtained from all patients. Both male and female patients, those between 18 and 80 years of age and with the American Society of Anesthesiologists (ASA) physical status I to III were included in the study. Patients with an inadequate fasting status, previous upper gastrointestinal or abdominal surgery, emergency surgeries, esophageal motility disorders, presence of nasogastric (NG) drain tube, nephropathy, and pregnant patients were excluded. This prospective, observational comparative cohort study was conducted after obtaining approval from the Institute Ethics Committee (IEC), IGMCRI (IEC approval no. 11/192/IEC-25/PP/2019) and was registered in the Clinical Trial Registry of India (registration no. CTRI/2019/06/019888).

A total of 220 consecutive patients satisfying the inclusion criteria were recruited into the study and divided into two groups: diabetic patients (n = 110) in the diabetes mellitus (DM) group and non-diabetic patients (n = 110) in the non-diabetes mellitus (NDM) group. In the DM group, the duration of diabetes and the presence of symptoms suggestive of peripheral neuropathy and autonomic neuropathy were recorded. Fasting and postprandial blood glucose levels were noted in the DM group. All patients were advised to fast from 12 midnight on the day before surgery. All patients received tablet famotidine 20 mg at 6 am on the day of the surgery according to the institute protocol. After the verification of the fasting status, patients were shifted to the preoperative holding area, and standard monitoring devices such as those for pulse oximetry, electrocardiogram (ECG), and non-invasive blood pressure (NIBP) were attached and values monitored. Fasting duration was noted. Gastric ultrasonography (GUSG) was done by a senior consultant who had experience in performing sonographic assessment of the antrum and was blinded to the patient's diabetes history.

**Figure 1:**
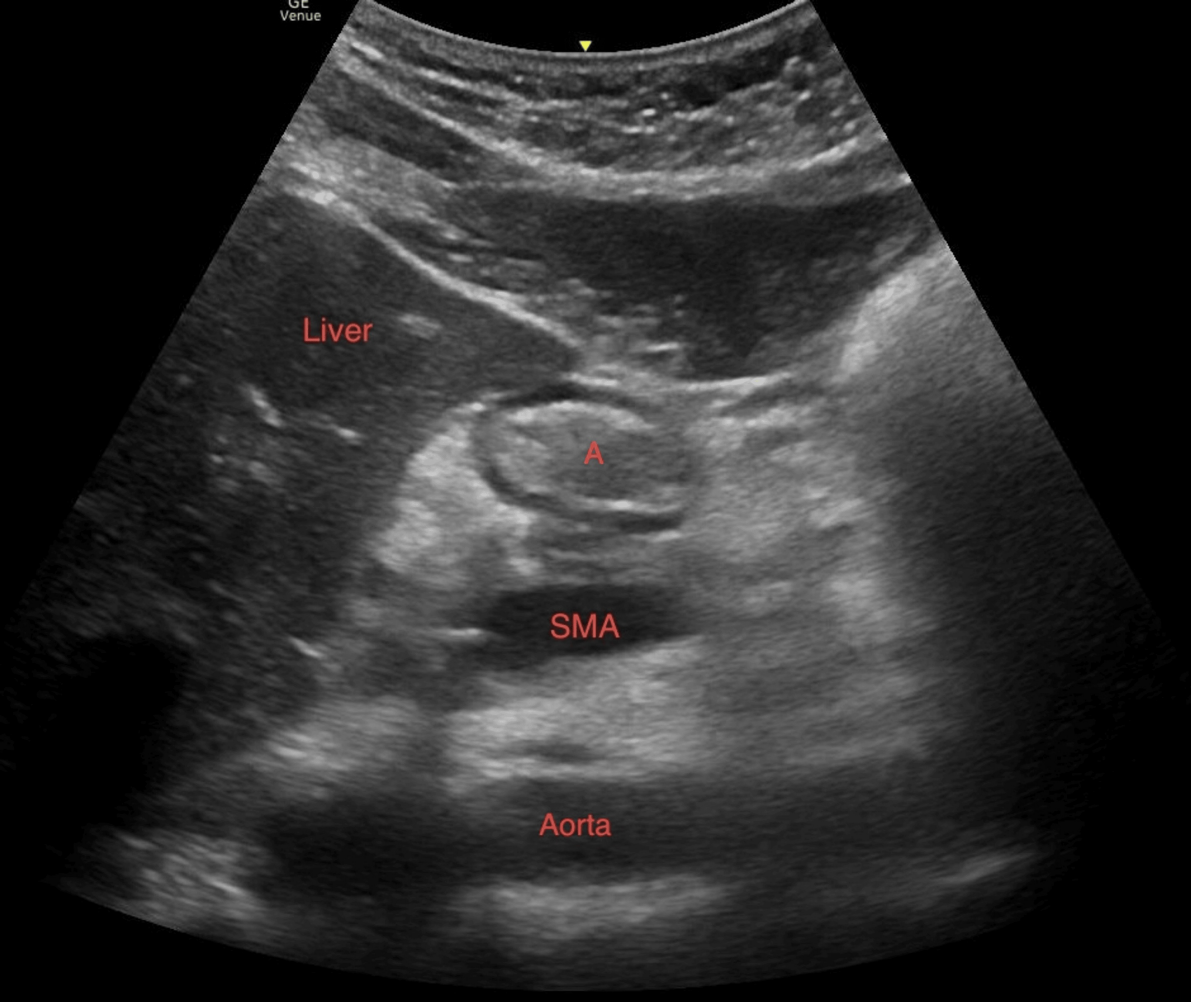
Sonographic image of the gastric antrum in the right lateral decubitus position A: antrum, SMA: superior mesenteric artery

GUSG was performed using a curvilinear ultrasound probe (2-5 MHz, M-Turbo; Fujifilm Sonosite, Inc., Bothell, WA, USA) in the parasagittal plane over the left upper abdominal quadrant in both the supine and the right lateral decubitus (RLD) positions. With the patient in the supine position, the ultrasound probe was placed in the midline of the upper abdomen in the sagittal plane and moved toward the left to identify the left lobe of the liver. Once the left lobe of the liver was visualized, the probe was changed to an oblique view to visualize the inferior vena cava or superior mesenteric artery. The gastric antrum was visualized either as a bull's eye pattern if there were no gastric content or a circular pattern if gastric contents were present (Figure 1).

The patient was placed in the right lateral decubitus position; the procedure was repeated and the findings were recorded. The antrum was classified into three grades: grade 0, with no fluid in the supine or RLD position; grade 1, with the presence of fluid only in the RLD position and grade 2, with the presence of fluid in the supine and the RLD positions or the presence of solids. The images were verified by another experienced anesthesiologist who was not part of the study. The antral cross-sectional area was calculated using the free tracing method in both supine and RLD positions (measured twice in each position). The predicted GV was subsequently calculated from the gastric antral CSA measured in the RLD position (mean of two values) by the formula proposed by Perlas et al. [5].

Patients with grade 2 antrum or GV >0.8 mL/kg or the presence of solid contents were considered as ‘high-risk’ antrum cases and to have a potentially increased risk of aspiration [6]. Patient details regarding USG findings of high-risk antrum were communicated to the respective consultant anesthesiologist and the anesthetic management protocol was followed at their discretion. Postoperatively, all study participants were followed up for 24-48 hours for the evidence of aspiration pneumonitis by a resident who was blinded to the study. The occurrence of fever, cough, breathlessness, pleuritic chest pain, and infiltrates seen on chest x-ray, if any, were considered suggestive of aspiration pneumonitis. The incidence of aspiration pneumonitis was noted.

The sample size was calculated considering the incidence of 3.5% for grade 2 antrum in fasting surgical patients and 14.3% in fasting diabetic patients [7]. With an alpha error of 0.05% and a power of 80%, and to allow for a 10% attrition, we calculated a sample size of 110 for each group. All the data were tested for normality using the Shapiro-Wilk test. Normal data were expressed as means and standard deviations and analyzed using the Student's t-test. Data not following normality were expressed as median and interquartile range and analyzed using the Mann-Whitney U test. Discrete variables were expressed as percentages and analyzed using the chi-square test. The association between patient variables in the diabetic group and a ‘high-risk’ antrum was further studied using univariate logistic regression to identify predictors of ‘high-risk’ antrum. The data were analyzed using IBM SPSS Statistics, version 24.0 (IBM Corp., Armonk, NY). A P-value less than 0.05 was considered statistically significant.

## Results

**Figure 2:**
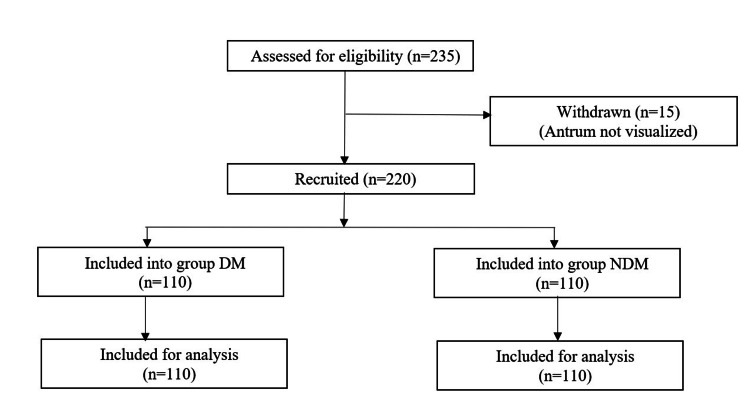
A STROBE diagram showing the study design DM: diabetes mellitus, NDM: non-diabetes mellitus, STROBE: STrengthening the Reporting of OBservational studies in Epidemiology

A total of 235 patients were assessed for eligibility for the study; 15 (10 in the DM group and 5 in the NDM group) patients were withdrawn due to difficulty in visualizing the antrum. The STROBE diagram depicting the study design is shown in Figure 2.

There were no significant differences in patient characteristics between both groups except for the higher ASA class in Group DM (Table 1).

**Table 1:** Baseline characteristics and fasting intervals DM: diabetes mellitus, NDM: non-diabetes mellitus, BMI: body mass index, ASA: American Society of Anesthesiologists, IQR: interquartile range Data are presented as median and IQR or number of patients. Medians (IQRs) were compared using the Mann-Whitney U test and proportions were compared using the chi-square test. A P-value <0.05 was considered significant.

	Group DM (n = 110)	Group NDM (n = 110)	P-value
Age, years	60 (53.7-66.2)	59 (53-65)	0.153
Gender (male/female), n	68/42	60/50	0.274
ASA (I/II/III), n	0/91/19	85/25/0	<0.001
BMI (kg/m^2^)	21.97 (20.07-24.22)	21/87 (18.27-23.61)	0.411
Fasting duration, h	11 (10-12)	12 (10.37-12)	0.260

Among the diabetic patients, 40% had symptoms of peripheral neuropathy, and 16% had symptoms suggestive of diabetic autonomic neuropathy. The median duration of diabetes was eight years (IQR, 3-12). The incidence of grade 2 antrum was significantly higher in diabetic patients (P<0.001). Antral CSA, GV, and GV (per kg) were significantly higher in group DM compared to NDM (Table 2).

**Table 2:** Gastric ultrasonographic findings for the two groups SD: standard deviation, IQR: interquartile range, DM: diabetes mellitus, NDM: non-diabetes mellitus Data are presented as means ± SDs, median (IQR) or number of patients (percentage). Means were compared using the paired t-test, medians were compared using the Mann-Whitney U test and percentages were compared using the chi-square test. ^*^P < 0.05. ^#^U-value. ^†^t-value. ^‡^Chi-square value.

Parameter	Group DM (n = 110)	Group NDM (n = 110)	Test statistics value	P-value
Antral cross-sectional area, cm^2^	5.65 (4.9-6.7)	4.67 (4.16-5.08)	2423.5^#^	0.000^*^
Gastric volume, mL	34.52 ± 20.02	21.63 ± 15.46	5.343^†^	0.000^*^
Gastric volume/weight, mL/kg	0.58 ± 0.33	0.37 ± 0.27	4.990^†^	0.000^*^
Grade, n (%)				
0	40 (36.6)	86 (78.1)	40.317^‡^	0.000^*^
1	50 (45.4)	20 (18.1)		
2	20 (18.1)	4 (0.03)		

A higher antral grade corresponded to higher CSA and GV (Table 3).

**Table 3:** Gastric ultrasonographic parameters in diabetic patients’ group SD: standard deviation, IQR: interquartile range Data are presented as means ± SDs and medians (IQRs). Means were compared using ANOVA; medians were compared using the Kruskal-Wallis test. *P < 0.05.

Grade	0 (n = 40)	1 (n = 50)	2 (n = 20)	P-value
Antral cross-sectional area, cm^2^	4.74 (4.12-6.87)	5.79 (5.22-6.55)	6.61 (5.66-7.34)	0.030^*^
Gastric volume, mL	27.28 ± 28.1	34.63 ± 17.97	45.79 ± 17.26	0.011^*^
Gastric volume/weight, mL/kg	0.44 ± 0.43	0.59 ± 0.33	0.84 ± 0.35	0.231

Diabetic patients were further classified into two groups: ‘high-risk’ and ‘intermediate/low-risk’ antrum for aspiration for subgroup analysis. Of the nine variables compared, gender and age were significantly different between the two groups (Table 4).

**Table 4:** Characteristics of diabetic patients with ‘high-risk’ and ‘intermediate/low-risk’ antrum SD: standard deviation, IQR: interquartile range Data are presented as means ± SDs, median (IQR) or number of patients (percentage). Means were compared using the paired t- test, medians were compared using the Mann-Whitney U test and percentages were compared using the chi-square test. ^*^P < 0.05. ^#^U-value. ^†^t-value. ^‡^Chi-square value.

Parameter	‘High-risk’ antrum (n = 41)	‘Intermediate/low-risk’ antrum (n = 69)	Test statistics value	P-value
Age, years	55 (51-64.5)	62 (55-67.5)	934.5^#^	0.003
Gender (male/female), n	19/22	49/20	6.633^‡^	0.015
BMI, kg/m^2^	21.78 (20.04-24.41)	22.03 (20.38-24.22)	1307.5^#^	0.508
Fasting glucose, mg/dL	95.44 ± 13.34	96.62 ± 13.64	-0.444^†^	0.658
Postprandial glucose, mg/dL	164.46 ± 32.9	162.84 ± 26.12	0.285^†^	0.776
Fasting duration, h	11 (10-12)	11 (10-12)	1296.0^#^	0.457
Diabetes duration, years	8 (4-12.5)	8 (3-12)	1348.5^#^	0.683
Peripheral neuropathy, n (%)	18 (43.9)	26 (37.6)	0.415^‡^	0.551
Diabetic autonomic neuropathy, n (%)	9 (21.9)	9 (13)	1.491^‡^	0.288

The univariate logistic regression model created showed that the incidence of a ‘high-risk’ antrum increased with the increasing age (OR = 1.06, P = 0.08), and a higher incidence was also associated with female gender (OR = 2.5, P = 0.015). There was no incidence of aspiration in the study population.

## Discussion

In this study, it was found that diabetic patients had a higher incidence of grade 2 antrum, and a higher CSA and GV compared to non-diabetic patients. It was also observed that female gender and increasing age were associated with a ‘high-risk’ antrum, potentially increasing the risk of aspiration.

Fasting allows the stomach to be devoid of any contents, so any gag reflex during intubation or extubation does not lead to aspiration, which is one of the most dreaded complications during the administration of anesthesia [8]. However, the conventional fasting guidelines do not always ensure an empty stomach, especially in situations of emergency surgery, pregnancy, and conditions like diabetes, which can delay gastric emptying [9]. POCUS has become unassailable in its utility to the modern anesthesiologist. This scenario of assessing gastric contents is no exception. The gastric antrum can be imaged in supine and RLD positions and based on the GUSG findings, can be qualified as grade 0, 1 and 2. In addition to qualitative assessment, quantitative assessment with prediction of the gastric volume will enable us to predict aspiration risk better [10,11].

Diabetic patients are frequently encountered in the anesthetic practice owing to the increased prevalence of metabolic syndrome and obesity in the current surgical population [12]. Since diabetic patients are prone to neuropathy-induced gastroparesis, they might be at a higher risk of aspiration [13]. POCUS assessment of the gastric antrum becomes very valuable in assessing the gastric contents in this scenario [9]. Multiple studies that evaluated gastric emptying either by sequential ultrasonography or by scintigraphy confirmed that gastric emptying is prolonged in patients with diabetes [14]. Hence, ultrasonographic estimation of antral grade and estimated gastric volume would be indispensable to assess the risk of aspiration in this group of patients.

Imaging the gastric antrum and grading the antrum according to the contents can provide a quicker way to predict the risk of aspiration. A higher antral grade corresponds to a higher volume, and hence, visualizing a grade 2 antrum might be considered a high risk for aspiration [15]. In this study, grade 2 antrum was found in 18% of patients in the diabetic group compared to 3% in the non-diabetic group, which is statistically significant. In a study by Zhou et al., the incidence of grade 2 antrum was 44% in diabetic patients following a two-hour fast after clear fluids [16]. Though the presence of solid contents predicted a higher risk of aspiration, the quantity of fluid that needs to be present to increase the risk of aspiration is still contentious.

Multiple studies have evaluated the gastric antrum in diabetic patients and predicted the aspiration risk in this group, but with variable findings [16-19]. There is a paucity of studies evaluating preoperative fasting patients scheduled for elective surgery in real time. A scoping review found seven studies that assessed the gastric content and volume in diabetic patients. They observed that there was a significant variability in the GV obtained, and they concluded that further studies with adequate sample size are needed to evaluate the antral grade, predict GV in the diabetic population, and identify risk factors predisposing patients to aspiration [20].

In two studies from India, one study found very small gastric volumes (9 mL in diabetic patients and 2 mL in non-diabetic patients) [17]. Another study by Sharma et al. observed higher volumes (around 57.2 mL) in diabetic patients [18]. Though both studies documented statistically significant higher volumes in diabetic patients compared to non-diabetic patients, the findings were inconsistent. In the current study, the average volume in diabetic patients was 34.52 mL and was 21.63 mL in non-diabetic patients, which was statistically significant.

In a study where nasogastric tube aspiration and gastroscopy-guided aspiration were used to validate the volume measured using GUSG, it was documented that the volumes measured using GUSG correlated well with NG tube aspiration, especially when the contents were liquid [21]. In the presence of solid contents, they may not get aspirated through the NG tube and can yield variable results. GUSG was better at visualizing the solid contents even when they could not be aspirated [22].

Even with the ability to predict the risk, the GV value above which the aspiration risk truly increases is contentious. Several criteria have been used in the past. Roberts and Shirley observed that a volume of more than 0.4 mL/kg when aspirated caused aspiration pneumonitis [23]. But the study was performed in animal subjects, and the entire volume was directly injected into the trachea. A GRV >0.8 mL/kg was used by Bouvet et al. to predict an increased risk of aspiration [24]. Van de Putte and Perlas came up with the volume of 1.5 mL/kg, as normal gastric secretions usually exceeded this value [4]. But this is still debatable as the risk of aspiration is determined by several factors such as difficult mask ventilation, difficult intubation, surgical stress and labor-induced changes, in addition to the elevated GV. In the current study, only one patient belonging to the diabetic group had a volume of more than 1.5 mL/kg. Diabetic patients with solid contents, grade 2 antrum and a GRV >0.8 mL/kg were considered ‘high-risk’ antrum cases and to be at a potentially higher risk of aspiration. In the current study, 37% of diabetic patients had a ‘high-risk’ antrum.

Since the logistics of scanning all preoperative diabetic patients is not plausible, it is important to identify diabetic patients with an increased risk of aspiration. In this study, we found that there was an increased incidence of ‘high-risk’ antrum with female gender and increasing age. In a retrospective observational study, it was found that older patients had a greater CSA and GV [25]. This correlates with the finding from the current study, where increasing age was associated with a potentially higher risk of aspiration. In the study by Zhou et al., it was found that the presence of diabetes-related eye disease predisposed patients to a higher aspiration risk [15]. In another study, it was observed that the duration of diabetes correlated with the increased risk of aspiration [26]. However, it is to be noted that none of the patients had any actual episode of aspiration in any of the studies including the current study. It is also pertinent that neuropathy associated with diabetes causes gastroparesis, increasing the risk of aspiration, but none of the studies found any association between the presence of neuropathy and increased aspiration risk.

Further studies with objective tests done to identify the presence of autonomic neuropathy, such as Valsalva technique, postural blood pressure changes, deep breathing (minimum-maximum heart rate) and prolonged handgrip, might help determine this relationship between GUSG and aspiration risk. This study has some limitations as well. This study included only patients with type 2 DM. The presence of autonomic neuropathy was only assessed clinically, and diabetes-related eye disease was not evaluated.

## Conclusions

Diabetic patients have a higher incidence of grade 2 antrum and greater GV when compared to non-diabetic patients and hence are at a potentially higher risk of aspiration, with associated risk factors such as increasing age and female gender, as observed in this study. Preoperative GUSG can be considered in diabetic patients to assess the risk of aspiration and for the apt modification of the anesthetic technique to ensure patient safety.
